# Un migrant de 19 ans consulte pour une urétrite et une éruption vésiculeuse

**DOI:** 10.48327/mtsi.v3i3.2023.376

**Published:** 2023-09-04

**Authors:** Patrick HOCHEDEZ, Paul-Henri CONSIGNY, Agnès DURAND, Pierre BUFFET

**Affiliations:** 1 Institut Pasteur, Centre médical, Paris, France; 2 Laboratoire d'analyses médicales Cerballiance, Paris, France

**Keywords:** Monkeypox, Varicelle, Éruption vésiculeuse, Lymphadénopathie, Migrant, Monkeypox, Chickenpox, Vesicular rash, Lymphadenopathy, Migrant

## Abstract

RÉSUMÉ Nous rapportons le cas d'un patient malien de 19 ans, ayant présenté une urétrite et une éruption vésiculeuse au cours de l’été 2022, dans les suites d'un probable rapport hétérosexuel. Le contexte épidémique parmi la population homosexuelle masculine et le tableau clinique sans atteinte génito-anale ni adénopathie font discuter une varicelle et un mpox, ce dernier étant finalement confirmé par la détection de l'ADN du virus Monkeypox sur du liquide vésiculaire.

## Abstract (en)

We report the case of a 19-year-old Malian patient, who presented with urethritis and a vesicular rash during the summer of 2022, following a probable heterosexual intercourse. The epidemic context among the male homosexual population and the clinical picture without genital lesions or lymphadenopathy allowed us to discuss both chickenpox and mpox, the latter being finally confirmed by the detection of Monkeypox virus DNA from vesicular fluid.

## CAS CLINIQUE

**Figure 1:**
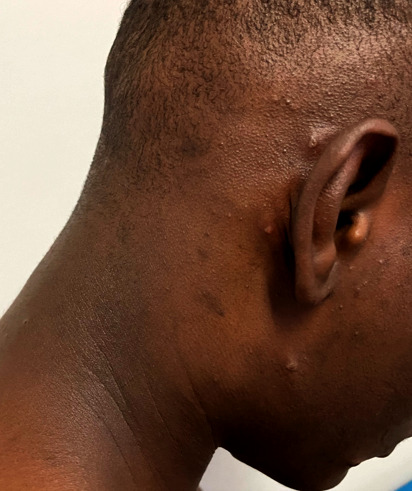
Lésions vésiculaires autour de l'oreille droite Vesicular lesions around the right ear

Au cours de l’été 2022, un Malien de 19 ans se présente dans un service d'urgences à Paris pour une sensation de brûlure à la miction, des lésions cutanées et une otalgie du côté droit. Il n'a pas d'antécédents médicaux significatifs, à l'exception d’épisodes de paludisme dans l'enfance. Le patient a quitté le Mali deux ans auparavant, il étudie à Paris et réside dans un foyer d'accueil. Les symptômes de dysurie et d’écoulement urétral purulent auraient commencé 2 jours avant la consultation, en même temps qu'une éruption vésiculeuse. L'examen clinique aux urgences met en évidence un écoulement urétral, six lésions vésiculeuses cutanées et une lymphadénopathie inguinale droite douloureuse. Les lésions sont situées respectivement sur l'abdomen, le dos et le bras droit, et trois lésions sont situées autour de l'oreille droite (Fig. 1). Le reste de l'examen clinique est sans particularité. Le patient nie initialement tout rapport sexuel récent, puis rapporte finalement des rapports sexuels non protégés avec une partenaire féminine, mais le délai entre les rapports et la survenue des symptômes ne peut être précisé. Il reçoit un traitement associant ceftriaxone parentérale et azithromycine orale (les deux en dose unique) pour suspicion d'urétrite à gonocoque et/ou *Chlamydia*.

Deux jours plus tard, il se présente à la consultation de maladies infectieuses. L'examen clinique n'identifie aucune nouvelle lésion cutanée, ni aucune lésion génitale ou périanale. Aucune adénopathie n'est observée, y compris dans la région inguinale où elle avait pourtant été précédemment décrite.

Un bilan sérologique (VIH, hépatite B, hépatite C et syphilis) et des prélèvements des lésions cutanées vésiculeuses sont réalisés pour analyse microbiologique. Le dépistage sérologique du VIH, de l'hépatite C et de la syphilis est négatif, et le patient a un profil sérologique d'hépatite B ancienne guérie. La recherche de *Chlamydia* et de gonocoque dans le 1^er^ jet urinaire est négative.

À ce stade, les deux principales hypothèses concernant cette éruption vésiculeuse sont une infection au virus Monkeypox (mpox) et une varicelle. La détection de l'ADN du virus varicelle-zona sur un prélèvement de vésicule s'est révélée négative, tandis que la détection de l'ADN du virus Monkeypox était positive (Monkeypox Virus Real Time PCR Kit, Bioperfectus^**®**^). Le patient a été contacté par téléphone avec recommandation de s'isoler pour une durée totale de 21 jours. Au cours de la période de son suivi, le patient n'a pas été au courant d'autres cas d’éruption dans son centre d'hébergement, et l'Agence Régionale de Santé n'a pas identifié d'autres cas au cours du mois suivant le diagnostic.

## Discussion

Cette observation clinique atypique de mpox survenue à Paris chez un jeune migrant de 19 ans, a priori à la suite d'une relation hétérosexuelle, s'intègre dans le contexte épidémique mondial évoluant depuis mai 2022, pendant lequel plus de 87 000 cas de mpox ont été notifiés à l'OMS par 112 États membres dans les 6 régions de l'OMS, la plupart dans la région des Amériques (68%) et la région européenne (29%), sans lien épidémiologique direct avec des zones d'Afrique de l'Ouest ou centrale [3]. À l'exception de ces pays d'Afrique, l’épidémie débutée en mai 2022 continue d'affecter essentiellement les hommes ayant des rapports sexuels avec des hommes (HSH), avec une dissémination principalement par le biais de réseaux sexuels. Selon l'OMS, parmi les cas pour lesquels des données sur l'orientation sexuelle étaient connues, 85% s'identifiaient comme HSH [3]. Les manifestations cutanées ont des caractéristiques spécifiques, avec une distribution inhabituelle des lésions, ce qui représente un changement significatif par rapport à la présentation classique de la maladie [4, 5]. Dans une étude sur 528 patients diagnostiqués dans 16 pays de quatre régions OMS (Europe, Amériques, Pacifique occidental et Méditerranée orientale), une transmission sexuelle était suspectée dans 95% des cas, et 98% des patients s'identifiaient comme HSH [5]. Dans cette série, presque toutes les personnes présentaient une éruption cutanée, 64% ayant moins de 10 lésions, 73% ayant des lésions anogénitales et 41% des lésions muqueuses (54 ne présentaient qu'une unique lésion génitale). Enfin, la majorité des patients présentaient de la fièvre (62%) et des adénopathies (56%). Dans une autre série de cas à Londres, les caractéristiques de la cohorte différaient à nouveau de celles des populations touchées lors de précédentes épidémies dans des régions endémiques [4]. Les 197 participants étaient tous des hommes et 196 s'identifiaient comme HSH. Tous présentaient des lésions cutanéo-muqueuses, le plus souvent sur les organes génitaux ou dans la région périanale, avec plus de 85% des participants signalant des signes généraux (fièvre et lymphadénopathies).

En dehors des nombreuses descriptions de mpox survenus chez les HSH en 2022, des cas ont aussi été rapportés chez des personnes ayant des rapports hétérosexuels occasionnels non protégés, comme au cours d'une étude réalisée au Nigeria entre juin et octobre 2022 [2]. Les auteurs rapportent le cas de 16 adultes atteints de mpox et ayant déclaré avoir eu des contacts sexuels avec des personnes atteintes de mpox confirmé en laboratoire dans le mois précédant le début de la maladie, en l'absence d'exposition antérieure à des animaux ou de contacts familiaux de mpox. La période d'incubation médiane était de 5 jours, et les 16 patients avaient une éruption génitale. Dans cette étude, la relation temporelle entre l'activité sexuelle et l'apparition des symptômes, la localisation des lésions dans la région génitale et l'absence d'expositions animales antérieures soutiennent la transmission interhumaine du virus par voie sexuelle.

Dans les pays endémiques, les manifestations cutanées du mpox ont souvent été décrites comme difficiles à distinguer des autres poxviroses, en particulier la variole et la varicelle. Depuis l’éradication de la variole, la varicelle demeure le principal diagnostic différentiel, et la présence de lymphadénopathies est un élément sémiologique distinctif clé en faveur du mpox, puisqu'elles ne sont pas typiquement décrites au cours de la varicelle (ou de la variole) [1]. Le patient que nous avons pris en charge n'avait ni fièvre ni lymphadénopathie lors de notre examen, une topographie d’éruption peu spécifique, sans lésion génitale ou périanale, et ne rapportait aucun rapport sexuel avec d'autres hommes. Devant cette présentation associée à une urétrite après un rapport hétérosexuel, et dans un contexte épidémique concernant surtout les HSH, nous avons donc considéré les 2 diagnostics, mpox et varicelle, et réalisé les prélèvements spécifiques, permettant d'obtenir un diagnostic de certitude.

## Conclusion

Depuis le début de l’épidémie de mpox débutée en mai 2022, la présentation clinique diffère considérablement de celle des populations touchées par les épidémies précédentes en zone endémique et non endémique, et les HSH sont principalement affectés. Devant un patient présentant une éruption vésiculeuse atypique, le clinicien doit essayer d'obtenir un prélèvement virologique cutané de confirmation diagnostique, indépendamment du nombre de lésions et de l'orientation sexuelle du patient.

## LIENS D'INTÉRÊTS

Les auteurs déclarent n'avoir aucun conflit d'intérêts.
